# Establishment and Validation of Sensitive Liquid Chromatography–Tandem Mass Spectrometry Method for Aldosterone Quantification in Feline Serum with Reference Interval Determination

**DOI:** 10.3390/ani15121687

**Published:** 2025-06-06

**Authors:** Tommaso Furlanello, Francesca Maria Bertolini, Andrea Zoia, Jose Sanchez del Pulgar, Riccardo Masti

**Affiliations:** 1 San Marco Veterinary Clinic and Laboratory, Via dell’Industria 3, 35030 Veggiano, Italy; tf@sanmarcovet.it (T.F.); francesca.bertolini@sanmarcovet.it (F.M.B.); zoia.andrea06@gmail.com (A.Z.); 2 CREA Research Centre for Food and Nutrition, Via Ardeatina 546, 00178 Rome, Italy; jose.sanchezdelpulgar@crea.gov.it

**Keywords:** cat, mineralocorticoid hormone, adrenal gland, LC-MS/MS

## Abstract

Aldosterone, a mineralocorticoid hormone synthesised in the adrenal cortex, is essential for maintaining electrolyte balance and fluid homeostasis. Its role in feline physiology remains underexplored, despite its importance in regulating sodium reabsorption and potassium excretion via mineralocorticoid receptors in renal tubules. This study is warranted given aldosterone’s importance in cats, particularly in light of their unique physiological traits, including highly concentrated urine and sensitivity to hydration status. Primary hyperaldosteronism, the most common feline adrenocortical disorder, contributes to arterial hypertension and chronic kidney disease, yet often remains underdiagnosed due to overlapping symptoms like hypokalaemia and hypertension. This research aimed to validate a liquid chromatography–tandem mass spectrometry (LC-MS/MS) method to measure serum aldosterone and to establish a reference interval in a population of healthy cats across a broad age range. The method demonstrated high precision and accuracy, with inter-assay coefficients of variation under 15%. Analysis of 49 healthy cats (40 young, 9 old) revealed a reference interval of 5.0–78.4 pg/mL (13.8–217.2 pmol/L). These findings provide a robust framework for diagnosing aldosterone-related disorders in cats and underscore the need for species-specific diagnostic tools. Improved understanding of aldosterone’s role could refine treatment strategies and enhance outcomes for affected feline patients.

## Simple Summary

Aldosterone is a crucial mineralocorticoid hormone in small animals, particularly cats, as imbalances can contribute to conditions like arterial hypertension, hypokalaemia, and chronic kidney disease. This study introduces a validated liquid chromatography–tandem mass spectrometry method for precise serum aldosterone measurement in cats, with a limit of quantification of 5 pg/mL. The method established a reference interval of 5.0–78.4 pg/mL (13.8–217.2 pmol/L) for healthy cats, providing a reliable tool for diagnosing and monitoring aldosterone-related disorders in veterinary practice.

## 1. Introduction

Aldosterone is a mineralocorticoid hormone synthesised in the zona glomerulosa of the adrenal cortex. It is essential for fluid balance, acid–base homeostasis, and electrolyte regulation [1]. Aldosterone primarily acts on the distal nephron, especially the principal cells of the connecting tubule and collecting ducts, to promote sodium reabsorption and potassium excretion. It also facilitates hydrogen ion exchange in intercalated cells, supporting systemic acid–base balance [2,3]. These mechanisms are particularly relevant in cats, given their ability to concentrate urine and their sensitivity to hydration changes [4,5].

Aldosterone deficiency is characteristic of hypoadrenocorticism, a rare feline disorder. Diagnosis often relies on cortisol measurement due to the greater availability of testing [6]. Conversely, accurate aldosterone quantification is critical for identifying primary hyperaldosteronism (PHA, or Conn’s syndrome), likely underdiagnosed in cats due to clinical similarities with common geriatric diseases. Most feline PHA cases stem from unilateral aldosterone-secreting adrenal tumours [7]. Excess aldosterone causes inappropriate sodium retention and potassium loss, leading to hypertension and hypokalaemia-induced polymyopathy.

Clinical signs include muscle weakness and hypertension-related ocular changes. Early symptoms may feature episodic forelimb stiffness and dysphagia, advancing to generalised weakness, plantigrade stance, poor jumping ability, and cervical ventroflexion. In severe cases, cats can develop flaccid paresis, muscle hypotonia, hyporeflexia, and respiratory compromise, complicating diagnosis and management [8]. The signs are often non-specific and overlap with diseases like chronic kidney disease and hyperthyroidism [8,9].

Steroid hormone measurement in veterinary medicine often uses immunoassays developed for human diagnostics, which lack full validation for feline samples. This limitation introduces variability and potential inaccuracies. Common methods—radioimmunoassay (RIA), enzyme-linked immunoassay (ELISA), and chemiluminescence immunoassay (CLIA)—are adapted for veterinary use but may be unreliable in cats due to species-specific biochemical differences [10,11,12]. Immunoassays are also prone to issues such as cross-reactivity, heterophile antibody interference, and the high-dose hook effect, reducing diagnostic accuracy [13].

These challenges are significant in feline PHA, where multiple steroid abnormalities often coexist. For example, one study found that over 30% of cats with PHA had serum progesterone levels ≥10 nmol/L (reference ≤2 nmol/L), and aldosterone levels above 3000 pmol/L were frequently associated with corticosterone concentrations ten times higher than in normal cats [14].

To address these limitations, liquid chromatography–tandem mass spectrometry (LC-MS/MS) is now considered the gold standard for steroid analysis, offering high specificity, sensitivity, and resistance to antibody-related interference [13,15,16,17].

This study aimed to validate an LC-MS/MS method specifically optimised for the measurement of serum aldosterone in feline samples. A secondary aim was to apply this technique to a reference population of healthy cats across a broad age range to establish species-specific reference intervals and characterise the physiological variability of aldosterone concentration in this population.

## 2. Materials and Methods

### 2.1. Serum Samples

Aldosterone concentration was measured using leftover serum samples from 49 cats presented between November 2023 and July 2024 to the San Marco veterinary clinic and diagnostic laboratory (Veggiano, Italy) for routine check-ups, with the aim of establishing reference intervals. Forty samples were obtained from healthy young cats, such as those undergoing initial vaccinations, neutering, first examinations, and retroviral testing, while the remaining nine were obtained from nine healthy adult cats. All cats were deemed healthy based on their medical history, physical examination (including arterial blood pressure measurement), and comprehensive diagnostic testing, which included a complete blood count, serum biochemistry, and urinalysis. No clinical evidence of dehydration or other potentially confounding factors known to influence aldosterone concentrations were observed. Blood samples were collected from the jugular, saphenous, or cephalic veins using clot activator-free tubes (Becton-Dickinson, Franklin Lakes, NJ, USA). The samples were centrifuged for 10 min at 3000× *g* to separate serum, which was subsequently transferred to plastic tubes, stored at −20 °C for a maximum of 2 months, and thawed immediately prior to analysis.

### 2.2. Chemicals and Reagents

Methanol hypergrade for LC-MS was purchased from LiChrosolv^®^ (Merck, Darmstadt, Germany). Aldosterone analytical standard (purity > 99.9%), D4-aldosterone internal standard, D4-cortisol, D8-cortisone and phosphate buffer (PBS) were purchased from Sigma-Aldrich^®^ (Merck, Darmstadt, Germany). Ammonium fluoride was purchased from Supelco (Merck, Bellefonte, PA, USA).

### 2.3. Instrumentation and Analytical Conditions

The analysis was conducted using an Agilent 1260 Infinity Series liquid chromatography (LC) system coupled with a 6490 Triple Quadrupole mass spectrometer (Agilent Technologies, Santa Clara, CA, USA). Sample separation was achieved on a Phenomenex Luna^®^ Omega Polar column (100 mm × 2.1 mm, 3 µm particle size; Phenomenex, Vaerloese, Denmark). The mobile phases consisted of 0.05 mM ammonium fluoride in water (Phase A) and pure methanol (Phase B). A gradient elution programme was applied as follows: 0–0.7 min at 40% B, 0.7–4.0 min linearly increasing from 40% to 70% B, and 4.1–5.0 min held at 100% B, followed by a re-equilibration at 40% B. The flow rate was maintained at 0.4 mL/min throughout the analysis, with an injection volume of 2 µL per sample. The total analysis time was 6.6 min. The column temperature was held constant at 40 °C, while the autosampler was maintained at 4 °C. The autosampler syringe was rinsed with a 50% methanol/water solution (*v*/*v*) between injections. The chromatographic system, including the column, gradient, and mobile phases, was optimised to achieve a retention time of 3.6 min, ensuring a retention factor (k′) greater than 2. The retention factor k′ was calculated using the formula: k’ = (tr−t0)/t0, where tr was the retention time of the analyte and t0 the dead time (void time). T0 was calculated using (column length × column diameter)/flow rate.

Electrospray ionisation (ESI) was operated in positive ionisation mode with the following source parameters: gas temperature at 180 °C, gas flow at 16 L/min, nebulizer pressure at 30 psi, sheath gas heater at 250 °C, sheath gas flow at 12 L/min, and a capillary voltage of 3500 V. Detection was performed using multiple reaction monitoring (MRM). For the quantification of aldosterone, the MRM transition *m*/*z* 361.2→315.2 was used as the quantifier ion, while *m*/*z* 361.2→97.2 served as the qualifier. The internal standard for aldosterone was monitored using the transition *m*/*z* 365.4→319.1 (quantifier), with *m*/*z* 365.4→347.2 and *m*/*z* 365.4→96.8 as qualifier ions. The fragmentor voltage was set at 380 V, and the collision cell accelerator voltage at 5 V. Collision energies were optimised to 15 eV for the transition 361.2→315.2, 18 eV for 361.2→97.2, 18 eV for 365.4→319.1, and 30 eV for 365.4→96.8. For the selectivity assessment, cortisone was monitored using the transitions *m*/*z* 361.2→298.9 (quantifier) and *m*/*z* 361.2→163.1 (qualifier). Cortisol was detected with transitions *m*/*z* 363.2→309.2 (quantifier) and *m*/*z* 363.2→97.1 (qualifier). The internal standard D8-cortisone was monitored using the transitions *m*/*z* 369.2→125.0 (quantifier) and *m*/*z* 369.2→169.0 (qualifier), while D4-cortisol was detected via *m*/*z* 367.2→121.1 (quantifier) and *m*/*z* 367.2→273.1 (qualifier). For all transitions used in the selectivity test, the collision energy was set to 15 eV. MassHunter Quantitative Analysis software version 6.0 from Agilent Technologies^®^ was used for quantitation and identification, while MassHunter Qualitative Analysis software version 6.0 from Agilent Technologies^®^ was used to identify the analytes.

### 2.4. Sample Preparation

The extraction procedure was performed as follows: a matrix-matched calibration curve prepared in 1X PBS with concentrations ranging from 5 pg/mL to 1 ng/mL was extracted alongside the samples. In total, 300 µL of serum samples was transferred into 1.5 mL microcentrifuge tubes, followed by the addition of 50 µL of a deuterated internal standard diluted in PBS at a concentration of 10 ng/mL. An extraction solution composed of Ethyl Acetate/Hexane (82.5:17.5 *v*/*v*) was added, and the mixture was vortexed for 5 min to ensure homogeneity. Phase separation was achieved through centrifugation at 30,000× *g* for 5 min, and the upper organic phase was carefully collected up to the meniscus and transferred to a separate 1.5 mL tube. The organic phase was evaporated to dryness under a gentle nitrogen stream. The residue was reconstituted with 100 µL of water/methanol (70:30 *v*/*v*) containing 0.10 mM ammonium fluoride, vortexed for 5 min, and subjected to ultracentrifugation at 30,000× *g* for 5 min. The sample was then transferred to vials and injected into the analytical instrument.

### 2.5. Method Validation

The method’s reliability and reproducibility were assessed following the European Medicine Agency’s Guideline on Bioanalytical Method Validation. Validation parameters included linearity, limit of quantification (LOQ), recovery, matrix effect, carryover, and stability (i.e., −20 °C freeze–thaw stability, room temperature 24 h stability, and 4 °C and −20 °C five- and seven-day stability). Additionally, intra- and inter-assay precision and accuracy were determined at four concentration levels: LOQ, low, middle, and high. The acceptance criteria required that the calibrator at the LOQ concentration deviated no more than ±20% from the nominal value, while calibrators at other concentrations remained within ±15% in each analytical run. The correlation coefficient (r^2^) was required to be ≥0.98.

#### 2.5.1. Standard Solutions and Calibration Curve

For the LC-MS/MS analysis, a stock solution of aldosterone and D4-aldosterone was prepared at a concentration of 1 µg/mL in methanol and stored at −20 °C to maintain stability. Because aldosterone is endogenously present in biological matrices, a truly blank serum was not available, so a surrogate matrix-matched calibration curve using PBS was employed. This curve was prepared in PBS to simulate the matrix environment and covered a concentration range from 5 pg/mL to 1 ng/mL. The surrogate calibration curve served to accurately represent the analyte’s quantification and ensured robustness and reliability in the absence of analyte-free biological matrices.

#### 2.5.2. Limit of Quantification, Linearity, and Carryover

The LOQ was determined empirically as the lowest concentration for which the method achieved a relative standard deviation (RSD) below 20% and a signal-to-noise ratio greater than 10. This was achieved by repeated analysis of the lowest calibration points, ensuring the method’s sensitivity met validation standards. Linearity was assessed through an eight-point calibration curve, which was matrix-matched to ensure accurate representation of the biological samples. The calibration curve was prepared and analysed on three consecutive days to confirm its reproducibility across analytical runs.

Carryover was assessed in conjunction with the linearity experiments by injecting three sequential blank samples immediately after the highest calibration point. The absence of detectable analyte signals in the blanks demonstrated that carryover effects were negligible and would not compromise the method’s reliability.

#### 2.5.3. Precision, Accuracy, Recovery, Selectivity and Matrix Effect

Precision, accuracy, and recovery were validated using a pooled set of six leftover serum samples, selected at random to ensure representativeness and sufficient volume for testing. These samples were spiked at four distinct concentration levels to evaluate the method’s performance across its analytical range. The concentrations used, according to the European Medicine Agency’s Guideline on Bioanalytical Method Validation, were 5 pg/mL (LOQ), 30 pg/mL (low, 3× LOQ), 500 pg/mL (medium, 50% of the calibration range), and 1 ng/mL (high, 100% of the calibration range). Each concentration level was analysed in five replicates across three non-consecutive days to determine both intra- and inter-assay precision. Precision results were expressed as the RSD, while accuracy was calculated as the percentage difference between the measured concentration and the nominal (expected) value. Recovery experiments involved spiking serum samples with known concentrations of aldosterone and comparing the measured responses to those obtained from standard solutions prepared in a solvent mixture of water/methanol (70:30 *v*/*v*) with 0.10 mM ammonium fluoride. This approach ensured a robust assessment of analyte recovery under the extraction conditions used. For the selectivity test, a blank sample was spiked with known concentrations of aldosterone, cortisol, and cortisone. To ensure accurate identification, internal standards D8-cortisone and D4-cortisol were added to aid identification. The matrix effect was evaluated by spiking post-extraction samples and comparing their responses to those of standard solutions prepared in water/methanol (70:30 *v*/*v*) with 0.10 mM ammonium fluoride. By quantifying the differences in analyte signal between the serum matrix and solvent-based solutions, the method’s robustness against matrix-related interference was confirmed. Coefficient of variation (CV%) values below 20% were set as the acceptance criteria for recovery and matrix effect evaluations. This comprehensive assessment of method validation parameters ensured the accuracy, reproducibility, and robustness of the analytical method for quantifying aldosterone in serum samples.

#### 2.5.4. Stability

Stability testing was conducted to assess the analyte’s robustness under varying conditions. Stability was evaluated using Quality Control (QC) samples at three levels: low (2× LOQ), intermediate, and high (near the upper LOQ). Three stock solutions at 10, 500, and 1000 pg/mL were prepared for analysis. Freeze–thaw stability was examined over three cycles of freezing at −20 °C and subsequent thawing at room temperature. Short-term stability was evaluated by maintaining samples at room temperature for 24 h, while long-term stability was assessed after 5 and 7 days of storage at 4 °C and −20 °C. For all stability assessments, acceptance criteria required that both the relative standard deviation (RSD) and the deviation from target values remained within ±15%, based on triplicate measurements at each concentration.

#### 2.5.5. Reference Interval

Reference intervals were established using data from all 49 feline samples.

#### 2.5.6. Statistical Analysis

Statistical analysis of the RIs was performed using Reference Value Advisor (V2.1) [18], a free software package for Microsoft Excel (2016). Potential outliers were identified using Dixon–Reed and Tukey tests, while the data distribution was assessed using d’Agostino–Pearson and Anderson–Darling tests, with statistical significance set at *p* ≤  0.05. Since the sample size was limited to 49 individuals, reference intervals were calculated using non-parametric methods, in accordance with the ASVCP 2011 guidelines, ensuring robust and clinically relevant RIs.

## 3. Results

### 3.1. Method Validation

The evaluation of linearity was conducted using a matrix-matched calibration curve ranging from 5 pg/mL to 1 ng/mL prepared in PBS. Calibration employed a non-forced linear regression with a 1/x^2^ weighting factor. The residual accuracy of back-calculated calibration points remained within ±15% of the nominal values, and the regression coefficient (R^2^) consistently exceeded 0.99. While limit of detection (LOD) was established at 2 pg/mL, the LOQ was established at 5 pg/mL, meeting criteria with a signal-to-noise ratio greater than 10 and a relative standard deviation below 15% across five replicates.

Precision and accuracy assessments were performed at four concentration levels: LOQ, low (3× LOQ), medium (50% of the calibration range), and high (100% of the calibration range). Intra- and inter-run precision and accuracy values for each concentration are summarised in Table 1. Inter-assay accuracy ranged between 94.5% and 102.2%, with inter-assay coefficients of variation (CV) remaining below 15% for all concentrations, including the LOQ. Matrix effect evaluation was performed in triplicate as previously described. The results, detailed in Table 1, demonstrate that the matrix effect was approximatively 100%. Carryover was evaluated following the injection of 1 ng/mL samples, and residual signals in subsequent blank samples were below 20% of the LOQ concentration. The internal standard exhibited carryover levels below 5%, ensuring compliance with analytical standards.

**Figure 1:**
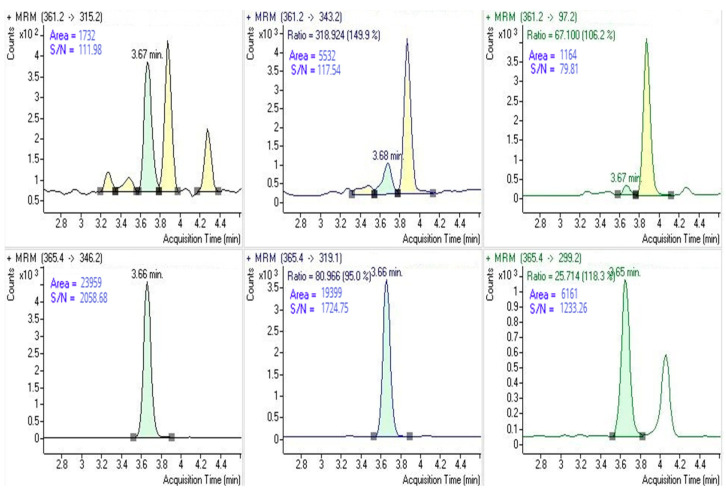
The top section of the figure displays the transitions used for aldosterone quantification along with their corresponding qualifiers. Below, the transitions utilised for quantification by the internal D4-aldosterone standard are shown, accompanied by their respective qualifiers. Image obtained from Agilent MassHunter Quantitative Analysis software, version 6.0 (Agilent Technologies, Santa Clara, CA, USA).

The retention time for aldosterone and D4-aldosterone is shown in Figure 1. The method’s specificity was demonstrated by the absence of interfering peaks at these retention times following the injection of the mobile phase and water. This was further confirmed by mass chromatograms, which showed no co-eluting endogenous compounds in the serum within the same time window.

#### 3.1.1. Selectivity

**Figure 2:**
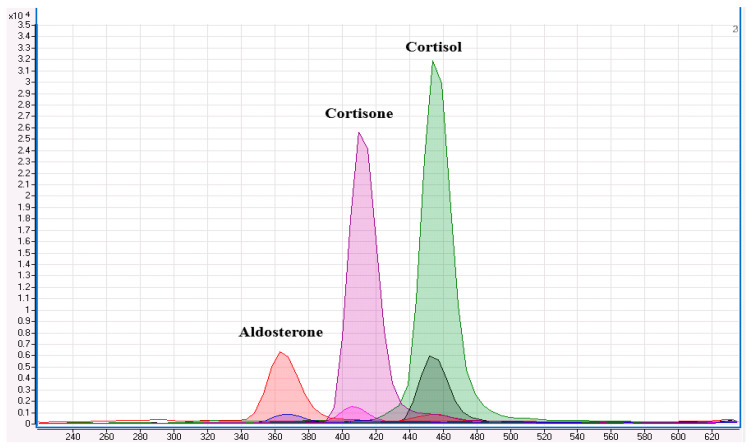
The chromatogram illustrates the separation and detection of three analytes along with their corresponding deuterated internal standards. From left to right, the peaks are assigned as follows: at a retention time of 3.6 min, the blue peak corresponds to aldosterone, while the red peak represents D4-aldosterone. At 4.1 min, the pink peak corresponds to cortisone, with the purple peak indicating D8-cortisone. At 4.6 min, the dark green peak denotes cortisol, and the light green peak corresponds to D4-cortisol. Image obtained from Agilent MassHunter Qualitative Analysis software, version 6.0 (Agilent Technologies, Santa Clara, CA, USA).

Given that aldosterone is part of a complex biosynthetic pathway involving structurally related steroids, a selectivity assessment was conducted as part of the method validation process. This evaluation aimed to identify potential co-eluting compounds with similar physicochemical properties that might interfere with aldosterone quantification and lead to overestimation. In particular, we investigated the potential interference from cortisone (an isobar of aldosterone) and cortisol, due to their structural similarities. To enhance analyte confirmation and ensure accurate identification, the corresponding stable isotope-labelled internal standards, D4-cortisol and D8-cortisone, were also included in the analysis. A matrix-matched calibration curve incorporating all analytes was prepared, and a blank matrix sample was spiked with a known concentration. As shown in Figure 2, chromatographic analysis demonstrates a clear resolution between aldosterone and the potentially interfering steroids. Despite the structural similarities and, in the case of cortisone, identical nominal mass, aldosterone was baseline separated from both cortisone and cortisol, confirming the method’s selectivity.

#### 3.1.2. Stability

Stability testing was performed on QC samples at low (2× LOQ), intermediate, and high (near ULOQ) concentrations. Freeze–thaw stability was assessed over three cycles at −20 °C and room temperature. Short-term stability was evaluated after 24 h at room temperature, and long-term stability after 5 and 7 days at 4 °C and −20 °C. Acceptance criteria were set as ±15% deviation and RSD.

Aldosterone showed acceptable stability under all tested conditions. Across freeze–thaw cycles, 24 h room temperature exposure, and prolonged storage at both 4 °C and −20 °C, all QC levels remained within ±15% of nominal values (Table 2).

### 3.2. Reference Interval

A total of 49 serum samples of clinically healthy domestic cats were included in the analysis for the determination of reference intervals. Of these, 40 were classified as young (aged 2 to 23 months; mean age: 9 months), comprising 21 males (11 intact, 10 neutered) and 19 females (11 intact, 8 spayed). The remaining nine cats were adult cats, consisting of three neutered males and six spayed females. The overall mean age of the study population was 5.6 years. The primary reasons for presentation among the young cats were routine screening for feline leukaemia virus (FeLV) and feline immunodeficiency virus (FIV) (*n* = 28), while the remaining 21 cats underwent general wellness examination or pre-neutering examination.

**Figure 3:**
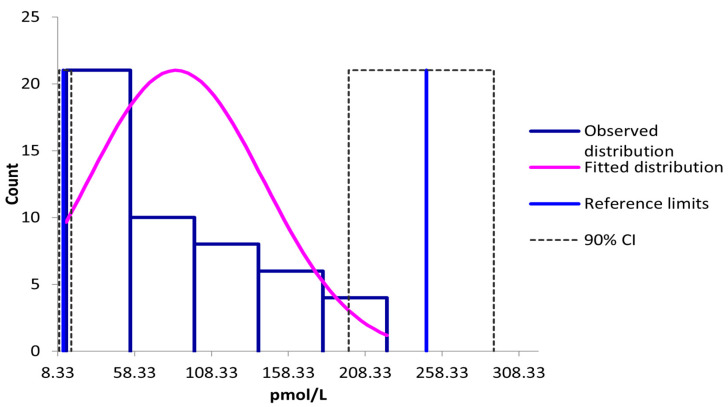
Distribution and reference intervals for aldosterone with Reference Value Advisor (V2.1) software [18]. CI denotes confidence interval.

Assessment of the distribution of the measured parameter was performed using the Shapiro–Wilk and Anderson–Darling tests, both of which indicated a non-Gaussian distribution (*p* < 0.01 and *p* < 0.05, respectively). The observed mean ± SD and median values were 30.7 ± 20.7 pg/mL and 24.5 pg/mL, respectively. Given the lack of normality, a non-parametric method was applied to establish the reference interval, yielding a range of 5.0 to 78.4 pg/mL. To align with the International System of Units (SI), concentrations were converted to pmol/L using a conversion factor of 2.774 [19], resulting in a reference interval of 13.8–217.2 pmol/L (Figure 3).

In light of the relatively limited sample size, a robust Box–Cox transformation was additionally performed to account for potential biological variability. This approach yielded a broader reference range of 4.3 to 89.5 pg/mL (equivalent to 11.9–248 pmol/L). However, as the lower limit of this interval fell below the assay’s quantification limit (5 pg/mL), the validated lower threshold of 5 pg/mL (13.8 pmol/L) was retained for the final reference interval.

## 4. Discussion

Clinical suspicion of primary hyperaldosteronism (PHA) in cats typically arises from a combination of signs, including systemic hypertension, retinal detachment, and hypokalaemia-induced muscle weakness, often manifesting as cervical ventroflexion or plantigrade posture [2,20,21]. Prompt diagnosis is important, as delayed recognition can lead to irreversible complications. Diagnosis generally relies on detecting elevated aldosterone levels alongside hypokalaemia, while imaging modalities such as ultrasound or CT scans may reveal adrenal abnormalities that support the diagnosis [9,22]. However, imaging availability and interpretation can be limited in routine practice, and the presence of adrenal masses does not always confirm functional aldosterone secretion, as non-secreting incidentalomas are common [21]. Moreover, PHA may occur without discernible adrenal enlargement, highlighting the need for reliable hormonal testing to confirm the condition [23]. These diagnostic limitations underline the importance of developing more reliable, accessible, and validated methods for measuring aldosterone in feline patients.

Currently, immunoassays remain the predominant method for measuring aldosterone concentrations in veterinary laboratories, owing to their relative affordability, automation compatibility, and rapid turnaround times. Nevertheless, the intrinsic limitations of immunoassays, including low sensitivity at lower hormone concentrations, susceptibility to cross-reactivity with structurally similar endogenous steroids and biologically inactive metabolites such as aldosterone glucuronide, and interference from heterophile antibodies and autoantibodies, can significantly compromise diagnostic accuracy [16]. In cats with concurrent renal disease, which is common in the geriatric feline population, these analytical challenges may be exacerbated, further complicating the differentiation between primary and secondary causes of hyperaldosteronism.

The emergence of LC-MS/MS offers a promising alternative. This technique enables direct molecular quantification, eliminating antibody-related cross-reactivity and improving analytical specificity [24,25,26]. In human endocrinology, studies have consistently shown that LC-MS/MS yields lower, and likely more accurate, aldosterone concentrations compared to immunoassays, revealing systematic overestimation by the latter, potentially skewing clinical interpretation [27,28,29,30]. Although human LC-MS/MS protocols are analytically robust, they frequently include complex sample preparation steps, such as extraction or derivatization procedures to enhance sensitivity [31]. Such complexity and associated costs limit their applicability in routine veterinary diagnostics. To date, no published LC-MS/MS protocols have specifically addressed the measurement of aldosterone in feline samples. Only a single study has mentioned aldosterone quantification in cats using LC-MS/MS, but it did not provide methodological details [32].

In response to this gap, the present study developed and validated a feline-specific LC-MS/MS protocol for serum aldosterone quantification, using a simplified liquid–liquid extraction approach designed to preserve analyte integrity while minimising matrix effects. The method showed high selectivity, effectively resolving aldosterone from structurally similar compounds such as cortisone, which may otherwise cause positive bias in conventional assays [33]. Common analytical interferences, including ion suppression/enhancement, in-source fragmentation, and co-eluting isobaric species, were evaluated using multiple transitions and internal standards, with minimal impact observed on assay reliability. The method was optimised to maximise analytical specificity and minimise in-source fragmentation, which can compromise quantification accuracy. MRM transitions for aldosterone were selected based on product ion scans identifying high-abundance, structurally specific fragments. Transitions were carefully evaluated to exclude low-mass or non-specific ions susceptible to matrix interference or in-source decay. Chromatographic separation and dwell time optimisation ensured consistent signal-to-noise ratios across the analytical range. Quantification employed a deuterated internal standard, closely matching the physicochemical properties of aldosterone. This isotopically labelled standard enabled accurate correction for matrix effects, ion suppression, and extraction variability, thereby enhancing both the precision and trueness of the assay.

The reference interval established from our population of healthy cats (5.0–78.4 pg/mL, or 13.8–217.2 pmol/L) was notably lower than values reported in earlier studies using radioimmunoassay (RIA), such as those by Javadi et al., where aldosterone concentrations ranged from 25 to 288 pg/mL in neutered cats and 4 to 205 pg/mL in intact cats [34]. While these earlier results provided foundational reference data, they should be interpreted with caution, as the RIA methodology lacks specificity for feline aldosterone and may overestimate concentrations due to cross-reactivity with inactive metabolites such as aldosterone-18-glucuronide and corticosterone. Additionally, the RIA protocol required approximately twice the sample volume used in the current study, which presents practical challenges in smaller or clinically fragile cats. Our results parallel findings in human medicine, where serum or plasma aldosterone levels measured by LC-MS/MS are consistently lower than those obtained with RIA [30], and are possibly due to the superior analytical specificity of the LC-MS/MS method due to its ability in direct molecular quantification with an improved analyte discrimination.

Adding to the variability in published data, Jepson et al. [35] investigated aldosterone levels in elderly hypertensive cats, stratifying subjects by azotaemia status and comparing them to healthy controls aged 11–14 years. Although informative, their methodology introduced further variability; notably, they used stripped human serum, depleted of endogenous steroids, as a matrix substitute during assay development due to the lack of feline-specific reagents. This substitution likely affected result accuracy, as differences in protein binding and metabolism between humans and cats may alter hormone measurements. This issue mirrors findings in human research, where RIA has been shown to overestimate aldosterone concentrations by approximately 29% compared to LC-MS/MS [29]. Such discrepancies have significant implications for feline diagnostics, potentially influencing clinical thresholds, case classification, and treatment decisions. The narrower aldosterone reference interval observed in healthy cats using LC-MS/MS supports the superior accuracy and analytical selectivity of this method, highlighting the limitations of immunoassay-based data. Clinically, a more precisely defined reference interval enhances the detection of subtle deviations in aldosterone concentrations, potentially improving the identification of early or subclinical PHA. Notably, borderline elevations that may have been considered physiological with RIA could potentially be recognised as pathological by LC-MS/MS. While definitive diagnostic thresholds for aldosterone concentration using LC-MS/MS in PHA diagnosis require clinical validation, our findings support re-evaluating the current RIA-based cut-offs for feline PHA [34]. Future studies incorporating sick cats and cats with PHA will be essential to establish more refined LC-MS/MS cut-off values for this condition.

The age distribution of the feline population used to calculate the reference interval was skewed toward younger cats (40 young vs. 9 old), and no analysis assessing age-related differences in aldosterone concentrations was conducted due to the low number of adult/elderly cats. Sex-based effects were also not evaluated, as subgroup analysis was beyond this study’s scope. Both factors merit further study due to their potential influence on mineralocorticoid activity. Additionally, sample collection occurred between November and July, excluding the hottest months when aldosterone secretion may vary with ambient temperature and hydration status. Although such variations are likely minimal in a healthy pet cat population with consistent access to water and shelter, future studies should aim for broader demographic representation and year-round sampling to better evaluate potential seasonal effects.

Several limitations should be acknowledged. First, although this method demonstrated strong analytical performance, clinical validation in cats with confirmed or suspected PHA is essential. The influence of common comorbidities, such as chronic kidney disease and systemic hypertension, on aldosterone levels in diseased cats requires further study. Second, despite the methodological simplification, LC-MS/MS remains less accessible than immunoassays for many veterinary settings due to higher costs and technical demands, which may restrict its widespread adoption outside referral laboratories. Third, because healthy cats may have aldosterone concentrations below the assay’s LOQ, basal aldosterone levels should not be used in isolation to screen for adrenal insufficiency or hypoaldosteronism. Finally, while serum sample stability was evaluated under certain storage conditions (i.e., 7 days), the longer storage duration at −20 °C used in this study (i.e., a maximum of 2 months) was not previously validated from us and may partially account for the unexpectedly low aldosterone values observed in this study’s healthy cohort. Nonetheless, previous studies have demonstrated that steroid hormones are generally stable over extended periods (e.g., 10 years) when stored under appropriate conditions at −20 °C [36,37]. Accordingly, it is reasonable to assume that the 2-months storage at −20 °C of our samples was relevant.

## 5. Conclusions

This study describes the first species-specific LC-MS/MS protocol for quantifying aldosterone in feline serum, offering an analytically robust and clinically viable alternative to traditional immunoassay-based methods. By providing enhanced specificity and sensitivity, this approach has the potential to improve diagnostic precision, support therapeutic decision-making, and ultimately advance outcomes in cats with adrenal disorders. While further work is needed to assess clinical utility, define diagnostic thresholds, and improve accessibility, this method represents a significant step toward more accurate and reliable endocrine diagnostics in feline medicine.

## Figures and Tables

**Table 1:** Accuracy of the determination of aldosterone, in terms of mean detected concentration. CV acceptance criteria for LOQ: ≤20%; CV acceptance for low, medium, and high levels ≤15%; EM% acceptance criteria: ≤20%.

Spike Level		Intra-Assay Bias 1	Intra-Assay Bias 2	Intra-Assay Bias 3	Inter-Assay Bias
LOQ (5 pg/mL)	Mean (pg/mL)	4.51	5.41	5.33	5.10
	CV	7.31	6.52	5.70	6.47
	Accuracy	90.2	108.4	106.5	101.7
Low (15 pg/mL)	Mean (pg/mL)	14.1	13.9	14.6	14.2
	CV	3.68	6.27	10.8	6.97
	Accuracy	94.0	92.7	96.8	94.5
Medium (500 pg/mL)	Mean (pg/mL)	524.3	511.6	497.1	511.0
	CV	2.14	2.26	3.16	2.51
	Accuracy	104.9	102.3	99.6	102.2
High (1000 pg/mL)	Mean (pg/mL)	1061.8	1003.7	991.6	1019.0
	CV	2.89	4.63	4.51	3.99
	Accuracy	106.2	100.4	99.2	101.9
EM %	Mean	99.1	-	-	-
	CV	1.72	-	-	-

Accuracy (%) = expressed as [(mean observed concentrations−nominal concentration)/(nominal concentration)] × 100); CV (%) = coefficient of variation: (standard deviation/mean) × 100. EM, matrix effect; LOQ, limit of quantification.

**Table 2:** The results of short-term stability (24 h at room temperature), long-term stability (5 and 7 days), and freeze–thaw stability (three cycles).

Stability Type	Nominal Concentration, pg/mL	Mean, pg/mL	Standard Deviation, pg/mL	Percentage Deviation
Short term	10	10.4	1.42	4.20
	500	467.3	10.1	−6.54
	1000	929.7	21.0	−7.03
Long-term refrigerated (5 days)	10	9.09	0.81	−9.10
	500	545.8	6.13	9.16
	1000	1066.7	30.4	6.67
Long-term freezing (5 days)	10	9.01	0.13	−9.90
	500	545.8	20.3	9.16
	1000	979.1	12.2	−2.09
Long-term refrigerated (7 days)	10	10.8	1.68	8.03
	500	546.5	59.16	9.29
	1000	1119.6	53.3	12.0
Long-term freezing (7 days)	10	10.54	0.99	5.39
	500	555.5	41.2	11.1
	1000	1043.7	22.7	4.37
Freeze–thaw	10	9.16	0.21	−8.39
	500	539.5	12.1	7.90
	1000	1044.8	27.6	4.48

## Data Availability

The dataset is available on request from the authors.
